# Chlorogenic acid attenuates pro-inflammatory response in the blood of streptozotocin-induced diabetic rats

**DOI:** 10.1186/s42826-022-00148-x

**Published:** 2022-12-02

**Authors:** Youngchan Lee, Chun-Sik Bae, Taeho Ahn

**Affiliations:** College of Veterinary Medicine, Chonnam National University, 77 Yongbong-Ro, Buk-Gu, Gwangju, 61186 Republic of Korea

**Keywords:** Chlorogenic acid, Cytochrome P450, Oxidative stress, Pro-inflammation

## Abstract

**Background:**

Chlorogenic acid (CGA) has been shown to reduce pro-inflammation by scavenging reactive oxygen species (ROS) and reactive nitrogen species. In this study, the anti-inflammatory effect of CGA was expanded to streptozotocin (STZ)-induced diabetic rats. The inter-relationships among oxidative stress, pro-inflammation, and cytochrome P450 (CYP) 1A enzymes were also investigated in peripheral blood mononuclear cells (PBMC) of STZ-diabetic rats.

**Results:**

The levels of pro-inflammatory cytokines, interleukin-6 and tumor necrosis factor-alpha, increased by approximately 3.4- and 2.9-fold, respectively, and the albumin concentration decreased in the serum of STZ-induced diabetic rats compared to normal rats. The C-reactive protein (CRP) values also increased by about 3.8-fold higher, indicating that STZ induced an inflammation in the blood of STZ-diabetic rats. The expression levels and catalytic activities of CYP1A enzymes were elevated by approximately 2.2–2.5- and 4.3–6.7-fold, respectively, in the PBMC of STZ-treated rats. A decrease in the amount of PBMC-bound albumin was also observed. In contrast, the levels of cytokines and CRP in serum and the activities of CYP1A enzymes in PBMC were significantly reduced in CGA-treated diabetic rats in a CGA concentration-dependent manner. In addition, STZ-mediated elevation of ROS in serum and PBMC was decreased by the CGA administration. However, the CGA treatment did not change the enhanced blood glucose level and expression of CYP1A enzymes by STZ. STZ-mediated decrease in the levels of serum and PBMC-bound albumin was not also restored by the CGA administration.

**Conclusions:**

These results suggest that CGA could be used to treat type 1 diabetes-induced inflammation.

## Background

Streptozotocin (STZ) has been used to induce type 1 diabetes mellitus (DM) in animal models. In STZ-treated rats, it was suggested that hyperglycemia, auto-oxidation of glycated protein, and the increased production of reactive oxygen species (ROS) elicit oxidative stress, which is accompanied by pancreatic β-cell damage [1]. In addition, STZ has been shown to deplete the cellular anti-oxidant pool, increasing the susceptibility of cells to oxidative damage [2]. Increased oxidative stress contributes to the etiology and pathogenesis of DM-associated chronic complications [3, 4].

Inflammation is a complex protective response against noxious stimuli, such as infection and cell injury, and is considered as a mechanism of innate immunity [5]. Inflammatory response involves various molecular mediators, endo- and exogenous inducers, and cellular effectors that cooperatively participate in the inflammatory pathway [6]. The mediators can be classified according to their biochemical properties: vasoactive amines, complements, lipid mediators, cytokines, and chemokines. ROS and reactive nitrogen species play an important role in the innate immune response to eliminate infectious agents [7]. However, it has also been well-known that the oxidative stress activates pro-inflammatory pathways and leads to chronic inflammation [8].

Chlorogenic acid (CGA) is a polyphenol and an ester formed between caffeic acid and quinic acid. CGA is the major active ingredient in some medicinal herbs and is also abundant in coffee beans, fruits (e.g., apples, grapes), and vegetables (e.g., carrots). It has been known that the scavenging of hydroxyl and superoxide radicals by CGA may protect against the oxidative stress-mediated cell injury [9]. It was also reported that CGA attenuates the generation of pro-inflammatory cytokines and thereby exerts anti-inflammatory activity [10]. Moreover, CGA has been shown to possess anti-bacterial and anti-carcinogenic properties [11], although its pharmacological mechanism in cells, except for ROS scavenging, is still unclear.

Cytochrome P450 (CYP) 1A enzymes, such as CYP1A1 and CYP1A2, are monooxygenases that metabolize drugs and xenobiotic molecules. CYP1A enzymes also contribute to the metabolic activation of pro-carcinogens, such as polycyclic aromatic hydrocarbons (PAHs) and heterocyclic aromatic amines [12]. Many studies have suggested that these enzymes play an important role in oxidative stress in animals because pro-inflammatory ROS and reactive metabolites are produced during the breakdown of PAHs and aromatic amines by CYP1A enzymes [13, 14].

In this study, the effect of CGA on the pro-inflammatory response in STZ-induced diabetic rats was investigated. In addition, we suggested the inter-relationships among oxidative stress, pro-inflammation, and CYP1A enzymes in the peripheral blood mononuclear cells (PBMC) of diabetic rats.

## Results

### Changes in body weight and blood glucose concentration

As a preliminary test, we first examined the clinical signs such as mortality and body weight. Treatment-related mortality and clinical signs were not observed in all rats tested during the study period (results not shown). In contrast to the gain in body weight observed in normal rats (control), the rats treated with STZ showed a significant decrease in body weight (Table 1). However, CGA treatments (CGA1: 10 mg/kg, CGA2: 50 mg/kg) after STZ administration resulted in a recovery of body weight, although statistic significances were not observed in the CGA1 group. Therefore, these results suggested that CGA may exert protective effects against the decrease in body weight induced by STZ. However, CGA treatments did not elicit any change in the STZ-induced increase in the blood glucose level (results not shown).

**Table 1:** Body weight changes in rats

	Group			
	Control	STZ	STZ/CGA1	STZ/CGA2
Before treatment	292.4 ± 13.2	296.1 ± 12.7	300.2 ± 9.4	295.9 ± 15.2
After treatment	301.3 ± 15.8	272.4 ± 11.5	279.5 ± 17.6	281.2 ± 18.9
Weight gain	9.5 ± 2.3	− 23.8 ± 4.2*	− 20.6 ± 7.9*	− 15.8 ± 5.5*,**

Values are presented as means ± S.D. of seven independent samples

*P < 0.05 compared with that of control, **P < 0.05 compared with that of STZ

### STZ-induced oxidative stress and pro-inflammatory response

To confirm that STZ could induce the pro-inflammatory response, including oxidative stress, in the blood of rats [15], we analyzed the biochemical parameters in the serum of STZ-diabetic rats. As shown in Table 2, the serum albumin levels were reduced by approximately 13%, and the concentration of hydrogen peroxide (H_2_O_2_) increased by approximately 2.2-fold in STZ-treated rats compared to normal rats (control). These results paralleled the previous suggestions that serum albumin was vulnerable to ROS generated during type 1 DM [16]. As anti-oxidant activity is one of the most prominent functions of albumin [17], the decreased albumin level may be responsible for the elevated production of H_2_O_2_. In addition, STZ treatment stimulated the production of H_2_O_2_ and lipid peroxidation (LPO) in the cytosolic and membrane fractions of PBMC by approximately 2.8- and 2.1-fold, respectively, compared to control. The STZ treatment also decreased the contents of albumin bound to PBMC (PBMC-albumin). These results collectively demonstrated that STZ administration was implicated in the enhanced generation of oxidative stress in both serum and PBMC, which was attributed to a decrease in the levels of serum albumin and PBMC-albumin [17, 18].

**Table 2:** Albumin concentration and oxidative stress in serum and PBMC

Assays	Group			
	Control	STZ	STZ/CGA1	STZ/CGA2
ALB/s	3.15 ± 0.34	2.73 ± 0.28*	2.64 ± 0.31*	2.87 ± 0.35*
ALB/p	1	0.68 ± 0.11*	0.61 ± 0.17*	0.64 ± 0.18*
H_2_O_2_/s	1.28 ± 0.62	2.82 ± 0.54*	1.63 ± 0.47**	1.32 ± 0.33**
H_2_O_2_/p	1.87 ± 0.82	5.35 ± 1.39*	3.75 ± 0.85*,**	2.21 ± 0.74*,**
LPO	3.47 ± 1.34	7.44 ± 2.86*	6.22 ± 2.45*,**	3.98 ± 1.82**

ALB/s, ALB/p, H_2_O_2_/s, and H_2_O_2_/p represent the levels of albumin and hydrogen peroxide in serum and PBMC, respectively

ALB/s: g/d*l*, H_2_O_2_/s: mM, H_2_O_2_/p: pmol/mg protein, LPO: nmol/mg protein

ALB/p was presented as relative amount of PBMC-bound albumin, compared to control, which was set to one

Values are presented as means ± S.D. of seven independent samples

*P < 0.05 compared with that of control

**P < 0.05 compared with that of STZ

**Fig. 1:**
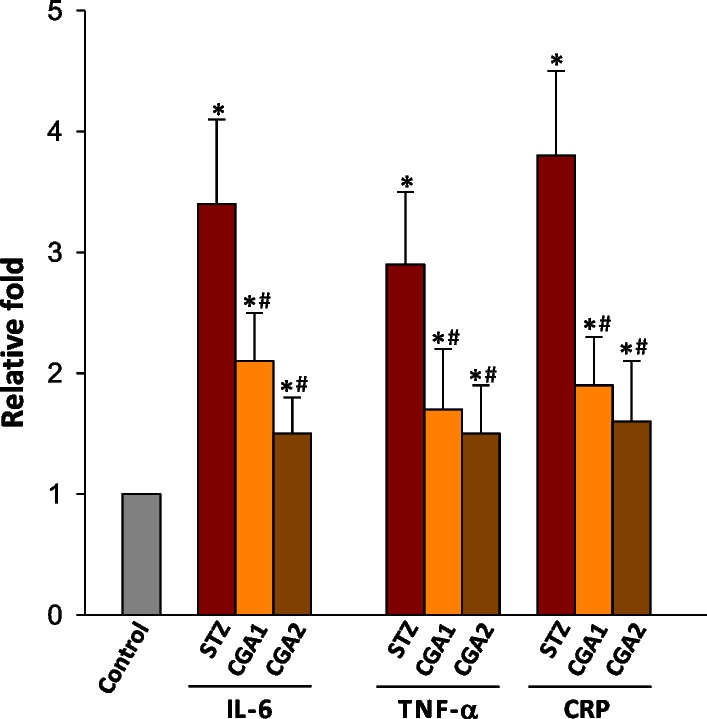
STZ- and STZ/CGA-induced changes in the levels of IL-6, TNF-α, and CRP. The relative concentrations of IL-6, TNF-α, and CRP in serum were measured from 1 ml blood of sample 1 week after treating rats with STZ or STZ plus CGA. The values of cytokines and CRP for untreated rat group (Control) were set to one (1). Values represent the mean ± S.D. of seven independent samples. *P < 0.05, compared to control and #*P* < 0.05, compared to STZ-treated rats, respectively

We investigated the status of the pro-inflammatory response in the serum of STZ-diabetic rats. The relative levels of interleukin-6 (IL-6) and tumor necrosis factor-alpha (TNF-α) in serum increased by approximately 3.4- and 2.9-fold, respectively, compared to the untreated rats (Fig. 1). Moreover, the serum concentration of C-reactive protein (CRP), a pro-inflammatory protein produced in the liver, was approximately 3.8-fold higher than control (Fig. 1). These results implied that STZ stimulates pro-inflammatory response and oxidative stress in type 1 DM rats, confirming the previous results [15]. It was also suggested that the plasma IL-6 and TNF-α were enhanced in STZ-induced type 2 DM rats [19]. However, changes in other parameters representing pro-inflammatory response were not systematically investigated in this study.

### Protective effect of CGA against ROS production and pro-inflammatory response

To examine the effect of CGA on ROS production and pro-inflammatory response in blood, two different concentrations of CGA was administrated to STZ-diabetic rats and the related parameters were measured. As a result, the levels of H_2_O_2_ and inflammatory cytokines tested including CRP in serum were significantly reduced by CGA administration in a CGA concentration-dependent manner, compared to those of STZ-diabetic rats (Fig. 1and Table 2). The concentration of H_2_O_2_ in CGA2 group was particularly comparable to that of the normal group, although the levels of IL-6, TNF-α, and CRP were still higher than those of normal rats. However, the concentrations of serum albumin and PBMC-albumin in the CGA1 and CGA2 group were similar to those of diabetic rats without CGA administration. Moreover, the CGA treatment did not restore the glutathione concentration (results not shown), although STZ has been known to decrease the reducing potentials in blood such as a reduced concentration of glutathione [18]. In addition to the reduction of H_2_O_2_ in serum, CGA2 group showed a decreased generation of H_2_O_2_ and LPO in the cytosolic and membrane fractions of PBMC, respectively, to levels similar to those of normal rats. These results suggested that CGA may alleviate STZ-induced ROS production and pro-inflammatory response in blood. However, the action mechanism of CGA should be revealed and the CGA concentration-dependent recovery effect against oxidative stress and inflammation is still unclear.

### STZ- and STZ/CGA-induced changes in the catalytic activities of CYP1A1 and 1A2

**Fig. 2:**
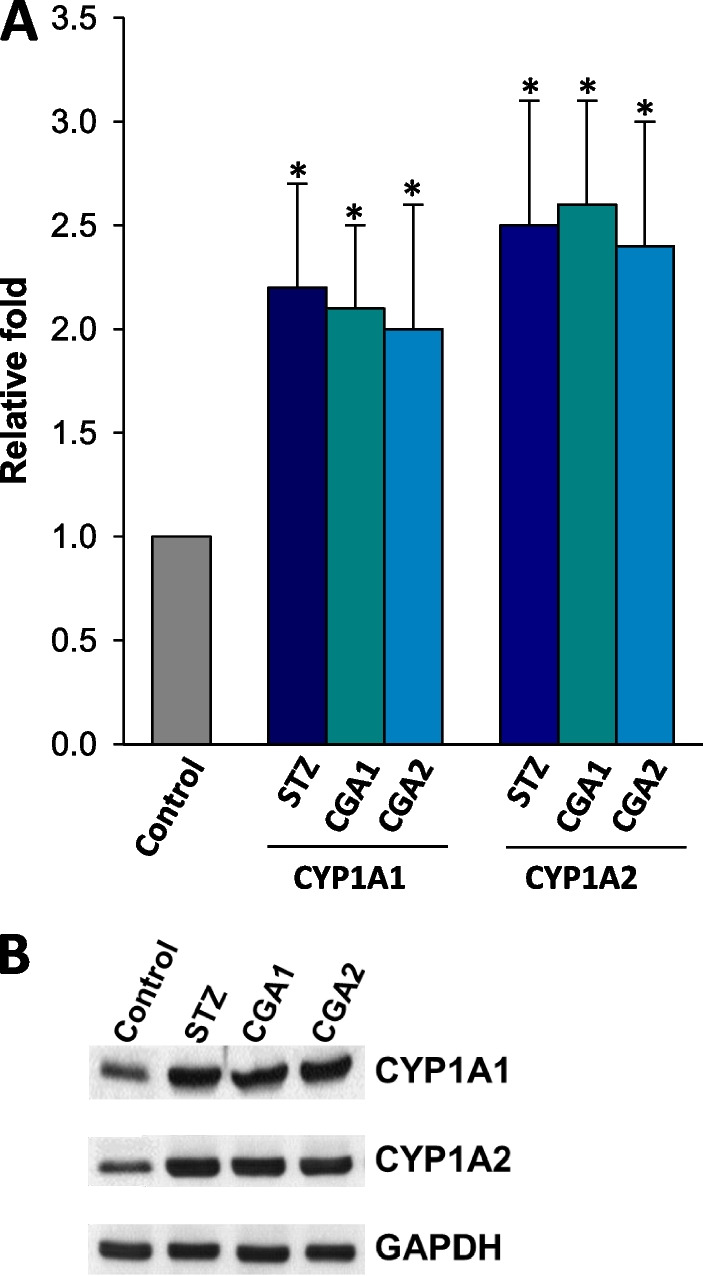
**A** qRT-PCR and **B** immunoblot analyses with RNAs and proteins, respectively, in PBMC. **A**: the transcriptional levels of CYP1A1 and 1A2 genes were expressed by relative fold change after the quantitation of cDNA. The transcription level of normal PBMC was normalized to be one (1) in the figure. Values represent the mean ± S.D. of seven independent samples. *P < 0.05, compared to control and #*P* < 0.05, compared to STZ-treated PBMC, respectively. **B**: immunoblot was performed with the protein extracts (50 μg/well) of PBMC, which were collected from seven independent samples. The ECL detection kit was used to visualize the protein bands

To obtain more insight into the relationship between oxidative stress and inflammatory response in blood, the expression of CYP1A enzymes in PBMC was analyzed in RNA and protein levels. These investigations were based on the previous results that CYP1A1 and 1A2 are expressed in peripheral blood leukocytes [20] and CYP1A enzymes can release ROS contributing to oxidative stress in cells [12, 21]. As the quantitative real-time polymerase chain reaction (qRT-PCR) results showed (Fig. 2A), the RNA levels for CYP1A1 and 1A2 increased by approximately 2.2- and 2.5-fold, respectively, in STZ-diabetic rat PBMC, compared to those of normal rats. The protein levels for CYP1A1 and 1A2 were also enhanced in the PBMC of STZ-treated group (Fig. 2B) by approximately 2.5-fold. These results suggest that STZ stimulated the gene expression of CYP1A enzymes in PBMC. However, any notable amount of RNA for cytochrome P450 2E1 (CYP2E1), another drug-metabolizing enzyme, which produces ROS as well [22], was not found in both normal and STZ-group when analyzed by qRT-PCR (results not shown).

**Fig. 3:**
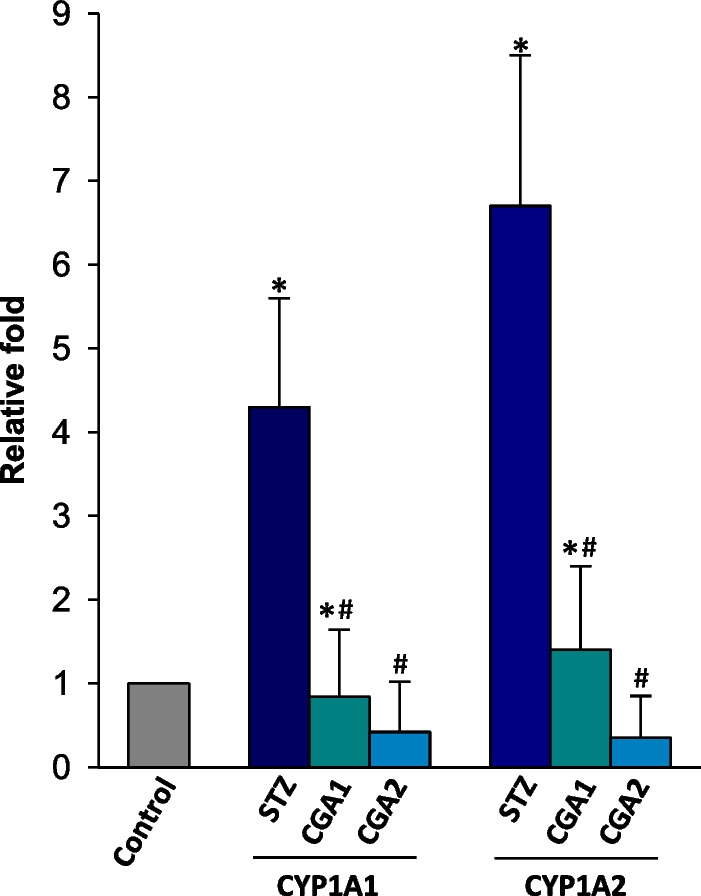
STZ- and STZ/CGA-induced changes in the catalytic activities of CYP1A1/1A2 in microsomes of PBMC. The enzyme activity of normal rat microsomes was set to be one (1) and the relative activities were expressed in the figure. Values represent the mean ± S.D. of seven independent samples. *P < 0.05, compared to control and #*P* < 0.05, compared to STZ-treated microsomes, respectively

In accordance with the enhanced expression of enzymes, the catalytic activities of CYP1A1 and 1A2 also increased by 4.3- and 6.7-fold, respectively, in STZ-diabetic rats, compared to those of normal rats (Fig. 3). Moreover, any prominent activity of CYP2E1 was not detected in PBMC when assayed by *p*-nitrophenol hydroxylation method. Measurement of the expression and catalytic activity of other types of CYP was beyond the scope of the present study, which focused on the effect of CGA on pro-inflammatory response, including the implication of CYP1A enzymes in PBMC.

### STZ- and STZ/CGA-induced changes in the expression of CYP1A1 and 1A2

In contrast to the effect of CGA on STZ-induced pro-inflammation and oxidative stress, CGA did not alter the expression levels of CYP1A1 and 1A2 in diabetic rats when measured by qRT-PCR and immunoblot analysis (Fig. 2A, B). However, the administration of CGA resulted in the reduced catalytic activities of both CYP enzymes, with almost complete inhibition of the both enzyme activities in CGA2 group. Taken together, it could be concluded that CGA acted as an inhibitor against CYP1A enzymes, but was not implicated in the enzyme expression. Nonetheless, it is unclear how CGA inhibited the activities of both CYP1A enzymes, and functional relevance of CYP1A1/1A2 in response to CGA treatment should be studied in more detail.

## Discussion

On the basis of the present results, it was not elucidated how CGA exerts anti-inflammatory effect in serum. However, it is anticipated that anti-oxidant properties of CGA contribute to its protective effect against STZ-mediated pro-inflammation in blood. This deduction is supported by the result in Table 2 that STZ decreased serum albumin level because serum albumin has an anti-oxidant activity and STZ depletes the cellular anti-oxidant pool, increasing the susceptibility of cells to oxidative damage [2, 17]. It was also suggested that low serum albumin levels are an indicator of the severity of inflammation [23]. Moreover, in the relation with the current study, it was demonstrated that CGA attenuates carbon tetrachloride-induced decrease in serum albumin level and thereby reduces liver inflammation in rats [24], although Table 2 showed marginal increase in serum albumin level by CGA2 treatment.

Taken together, the current results imply that CGA may act as a substitute of albumin, providing a protective effect against oxidative stress and resultantly attenuates pro-inflammation. However, any experimental evidence(s) supporting this notion has not been reported, although it was suggested that bovine serum albumin increases anti-oxidant effect of CGA in copper-induced oxidation of low-density lipoprotein [25]. Furthermore, the direct correlation between the administered CGA concentration and alleviation of STZ-induced pro-inflammation, including the degree of oxidative stress, is unclear. Based on the results that CGA could not restore a STZ-mediated decrease in the levels of serum albumin (and also PBMC-albumin) which scavenges ROS, it may be deduced that CGA itself acts as an anti-oxidant, as already suggested in hepatocytes [26]. In spite of these suggestions, however, the inter-relationships of serum albumin levels, oxidative stress, and pro-inflammation are still unknown.

IL-6 and TNF-α are mainly produced by monocytes and macrophages during acute inflammation. These inflammatory cytokines are synthesized and released from the innate immune cells through various triggers and signaling pathways. For example, IL-6 is secreted in response to specific pathogens, which bind to detection molecules, such as Toll-like receptors, on the cell surface and elicit intracellular signaling cascades. However, there is no distinct evidence that oxidative stress may induce the production and release of these pro-inflammatory cytokines, although oxidative stress directly induces the pro-inflammation progress [8]. On the contrary, it was reported that pro-inflammatory cytokines increase ROS and resultant cell damage [27]. While oxidative stress may not act as a direct inducer of the cytokine generation, it is accepted that ROS can affect or oxidize the cellular components in immune cells and thus promote the release of pro-inflammatory mediators including cytokines [28].

In relation with CGA-mediated regulation of pro-inflammatory cytokine synthesis, it was reported that CGA treatment decreases the gene expression of IL-6 and IL-8 in lipopolysaccharide-induced inflammation of IPEC-J2 cells [29]. It was also demonstrated that CGA reduces hepatic mRNA expression and serum levels of TNF-α, IL-6, and interleukin-1β in carbon tetrachloride-induced inflammation of rats [24]. Therefore, taken together with the present results, these reports provide the conclusion that CGA directly decreases the gene expression of pro-inflammatory cytokines and results in the attenuation of inflammation. However, the molecular mechanism(s) for CGA-mediated gene repression is unclear at present.

Based on the knowledge that CYP1A enzymes generate oxidative stress and oxidative stress induces pro-inflammatory response in cells, the current results imply that CGA exerts anti-inflammatory effect through its anti-oxidant activity and inhibition of functional CYP1A enzymes in PBMC. The present results further suggest that the enhanced expression of CYP1A1 and 1A2 may be responsible to the elevated production of H_2_O_2_ and LPO in serum and PBMC as the enzymes are known to generate ROS in cells. However, because the STZ-mediated origins of ROS are expected to be diverse, the contribution of CYP1A family in PBMC to serum ROS levels is unclear. These results parallel the previous result, suggesting that CGA ameliorated the acetaminophen-induced liver injury probably by inhibiting CYP1A2 enzymatic properties [30]. However, because the inflammation can be provoked by various triggers and oxidative stress is not a sole inducer of the inflammation, the present results do not explain how CGA contributes the protective effect against STZ-induced inflammation in the blood. As shown in Fig. 2, it is also unclear how STZ stimulates the expressions and activities of CYP1A1/1A2 enzymes in PBMC. Nonetheless, it could be anticipated that the inhibition of CYP1A activities may be implicated in the anti-inflammatory effect of CGA.

No distinct expression of CYP2E1 in the present results appears contrary to the previous reports that STZ elevated the expression and catalytic activity of CYP2E1 in the liver [31] and that CGA inhibited CYP2E1 activity in hepatic cell line [30]. Furthermore another study detected gene expression of CYP2E1, including CYP1A1/1A2 in PBMC and the liver from human patients with hepatocellular carcinoma [19]. This discrepancy may be caused by species differences in CYP genes [32]. However, because CYP2E1 produces ROS and CGA inhibits the enzyme activity [30, 33], the expression of CYP2E1 is important to elucidate the action mechanism(s) of CGA in pro-inflammation. Therefore, further study should be undertaken to verify the presence of CYP2E1 in rat PBMC. Moreover, it is still unclear how CGA attenuates CYP1A1 and 1A2 enzymatic properties. Based on the result from Fig. 3, CGA may act as an inhibitor against the catalytic activities of CYP1A enzymes, but not as a regulator of CYP1A gene expression. By contrast, it has been suggested that CGA activates a nuclear factor erythroid 2-related factor 2, a transcription factor regulating the expression of anti-oxidant enzymes, in *Caenorhabditis elegans* [34].

## Conclusions

In this study, we demonstrated that CGA could attenuate STZ-mediated pro-inflammatory response in rat blood, which may be attributed to a decrease in the elevated ROS by STZ. In addition, we suggested that CGA inhibited the activities of CYP1A enzymes without a change in STZ-induced stimulation of the gene expression and reduced ROS production in PBMC. All the current results collectively present the possibility that CGA may be used as a remedy to attenuate detrimental effects of type 1 diabetes on the inflammatory status in the blood.

## Methods

### Animals and environmental conditions

Seven-week-old male Sprague–Dawley rats, weighing 253.5 ± 14.7 g, were provided by Samtako Bio Korea Co., Ltd. (Osan, South Korea) and were used after 1 week of quarantine and acclimatization. The rats were housed in an air-conditioned room with a 12 h light/dark cycle under controlled illumination (200–300 lx), temperature (23 ± 2 °C), and humidity (55 ± 10%). Tap water and commercial rodent diet (Samyang Feed, Wonju, South Korea) were provided ad libitum. The animals were randomly divided into experimental groups (*n* = 7 per group) as follows: (1) normal (control) group; (2) STZ-treated group; (3) 10 mg/kg CGA-treated group (CGA1); (4) 50 mg/kg CGA-treated group (CGA2).

### Induction of DM and CGA administration

Diabetes was induced by a single intraperitoneal injection of STZ (100 mg/kg body weight in 0.1 M citrate buffer). Rats in the non-diabetic group (control) were injected with equivalent volume of 0.1 M citrate buffer only. One week after the injection, severity of the induced diabetic state was assessed by monitoring blood glucose levels using reagent strips (ACCUTREND, Roche Diagnostics GmbH, Mannheim, Germany). Rats with blood glucose levels exceeding 300 mg/d*l* were classified as diabetic rats. Bloods were collected from the caudal vena cava of each rat. CGA was purchased from Sigma-Aldrich (St. Louis, MO, USA). CGA were administered orally at a concentration of 20 mg/m*l* once per day for 6 days, with the first administration 1 h after STZ injection.

### Blood biochemical analyses

The animals were anesthetized with a combination of xylazine hydrochloride (Rompun; Bayer Korea, Seoul, South Korea; 10 mg/kg) and ketamine HCl (Yuhan Co., Seoul, South Korea; 40 mg/kg) and blood samples were collected by venipuncture from the posterior vena cava. Each sample was centrifuged at 1000 × *g* for 15 min within 30 min after collection and then the top serum layer was removed. The amounts of albumin in serum were assayed using an autoanalyzer (Dir-Chem 4000i, Fujifilm, Tokyo, Japan) by standard methods. Dedicated ELISA assay kits were used to measure the serum levels of IL-6 and TNF-α (Sigma-Aldrich) and CRP (Abcam, Cambridge, UK).

### Isolation of PBMC

PBMC were prepared from whole blood using as described previously [17]. Briefly, EDTA-treated blood was diluted 1:1 with PBS containing 2% fetal bovine serum, and layered onto Ficoll-Paque PLUS (Sigma-Aldrich) at a 2:1 ratio of blood to PBS:Ficoll. The blood was centrifuged at 400 × *g*, 25 °C for 40 min. The buffy coat layer was removed and washing steps were performed. The cells were suspended in a balanced salt solution (provided by the manufacturer), and then the sample was centrifuged at 100 × *g*, 25 °C for 10 min. The pellet was collected after removal of supernatant and this washing step was repeated twice. The isolation of PBMC from rat blood was verified by flow cytometry analysis using anti-pan B-cells antibody (clone number of 68-IB3, Santa Cruz Biotechnology, Dallas, TX, USA). The concentration of live PBMC was assayed using a 3-(4,5-dimethylthiazol-2-yl)-2,5-diphenyltetrazolium bromide (MTT) assay kit (Sigma-Aldrich) in accordance with the manufacturer’s instructions. After isolation of PBMC from 1 ml blood, MTT solution was added to the cells in 96-well plates. After the incubation of the plates at 37 °C for 3 h, the required volume of solubilizing solution was added and the absorbance at 590 nm was measured. The amounts of albumin in PBMC were determined by the method as described previously [17].

### Measurement of oxidative stress

The amount of H_2_O_2_ in serum was measured spectrofluorimetrically using the Amplex Red (AR) assay kit (Invitrogen, Carlsbad, CA, USA) according to the manufacturer’s instructions. Flurescence was recorded at 571 nm excitation and 585 nm emission wavelengths. The cytosolic and microsomal fractions of PBMC were obtained using a previously described method [35]. LPO in the membranes of PBMC was estimated by measuring thiobarbituric acid-reactive substances, using malondialdehyde as a standard, as described previously [36].

### CYP activity and immunoblot assays

CYP1A enzyme activities were measured using P450-Glo CYP1A1 Assay and CYP1A2 Assay (Promega, Madison, WI, USA). To measure the activity of CYP2E1, the *p*-nitrophenol hydroxylase activity was measured as reported [37]. For all assays of CYP enzymes, microsomal fractions of PBMC were used. Total protein concentrations were determined using Bradford assay with bovine serum albumin as the protein standard. The protein extracts (50 μg/well) were separated by SDS-PAGE using 11.5% polyacrylamide gel. After electrophoresis, immunoblot analysis was performed using a conventional method with an anti-CYP1A1 or 1A2 antibody [Abcam, 1/1,000 (v/v)]. Proteins were visualized using the enhanced chemiluminescence (ECL) (Amersham Biosciences, Buckinghamshire, UK). Protein band intensities were quantified using ImageJ software (National Institute of Health, USA).

### qRT-PCR

Total RNA was extracted from intact PBMC (approximately 2 × 10^6^ cells) using the RNeasy Mini Kit (Qiagen, Hilden, Germany). The extracted RNA was used to synthesize first-strand cDNA using the Reverse Transcription System (Promega) according to the manufacturer’s protocol. qRT-PCR was performed using Rotor-Gene 6000 (Qiagen), and the 2^−ΔΔC^T method was used for relative quantitation. Threshold cycle number and reaction efficiency were determined using Rotor-Gene 6000 series software version 2.7. The primers for CYP1A1, CYP1A2, CYP2E1, and glyceraldehyde 3-phosphate dehydrogenase (GAPDH) genes were obtained from OriGene (Rockville, MD, USA).

### Statistical analysis

Statistical data were expressed as the mean ± standard deviations (S.D.) of at least seven samples. One-way ANOVA computed by SPSS version 19.0 (SPSS Inc., Chicago, IL, USA) was used for statistical analysis and a *P*-value < 0.05 was considered significant.

## Data Availability

The datasets used and/or during the current study are available from the corresponding author.

## Abbreviations

CGA: Chlorogenic acid
CRP: C-reactive protein
CYP: Cytochrome P450
DM: Diabetes mellitus
GAPDH: Glyceraldehyde 3-phosphate dehydrogenase
IL-6: Interleukin-6
LPO: Lipid peroxidation
PBMC: Peripheral blood mononuclear cells
ROS: Reactive oxygen species
qRT-PCR: Quantitative real-time polymerase chain reaction
STZ: Streptozotocin
TNF-α: Tumor necrosis factor-alpha
